# An open, retrospective study on the duration of virus shedding for Shanghai patients infected with SARS‐CoV‐2 omicron variants

**DOI:** 10.1002/hsr2.1088

**Published:** 2023-01-31

**Authors:** Hai Zou, Jun Zhang, Wencong Chen, Xinyan Li, Biao Zhu

**Affiliations:** ^1^ Department of Critical Care Fudan University Shanghai Cancer Center Shanghai China; ^2^ Department of Oncology Shanghai Medical College, Fudan University Shanghai China; ^3^ Department of Internal Medicine LongHua Hospital Shanghai University of Traditional Chinese Medicine Shanghai China; ^4^ Department of Biostatistics Vanderbilt University Medical Center Nashville Tennessee USA; ^5^ Department of Hepatology Shanghai Public Health Clinical Centre, Fudan University Shanghai China

**Keywords:** ALC, COVID‐19, duration of virus shedding, erythrocyte sedimentation rate, SARS‐CoV‐2 infection nucleic acid testing

## 1 INTRODUCTION

According to the Shanghai Municipal Health Commission, as of May 4, 2022, there were 601,942 COVID‐19 patients in Shanghai, among which 547,056 were asymptomatic.^1^ The viral genomes responsible for recent infections belonged to the SARS‐CoV‐2 BA.2 subtype; the Omicron strain has lower rate of critical illness and mortality than the previous Delta strains.^2^, ^3^ Despite its low toxicity and mild symptomatology in most patients, this condition largely affects society, economy, work, and life. The main criterion for determining patients' recovery and release from quarantine is a negative SARS‐CoV‐2 nucleic acid test result. Therefore, the conversion of tests from positive to negative is highly significant for mildly symptomatic COVID‐19 patients, with respect to both their treatment and their social lives after discharge. This study aimed to identify the clinical features and laboratory indicators that correlate to negative conversion time, allowing for early prediction of this important factor.

## 2 METHODS

This retrospective study included inpatient data of patients with mild COVID‐19 admitted to the Shanghai Public Health Clinical Research Center from April 1, 2022, to May 1, 2022. All cases were confirmed COVID‐19‐positive by reverse transcription polymerase chain reaction (RT‐PCR) using nasal and/or throat swab specimens. All patients were vaccinated.

The patients' clinical data were extracted from the hospital's electronic medical records. The baseline patient characteristics included the cycle threshold (Ct) values of the initial RT‐PCR detection results (ORF1a/b, N gene); nucleic acid conversion time; age; sex; body mass index (BMI); underlying diseases including diabetes, hypertension, chronic obstructive pulmonary disease, and coronary heart disease; and laboratory examination indicators: white blood cell‐, neutrophil‐, platelet‐, and lymphocyte counts, neutrophil‐to‐lymphocyte‐ and platelet‐to‐lymphocyte ratios, hemoglobin, albumin, prealbumin, alanine aminotransferase, aspartate transaminase, serum creatinine, serum uric acid, d‐dimer, high‐sensitivity C‐reactive protein, serum amyloid A, interleukin 6 (IL‐6), interleukin 8 (IL‐8), CD3+ T lymphocyte count, CD4+ T lymphocyte count, and CD8+ T lymphocyte count. All indicators were detected within 2 days after admission and were included in the study as predictors.

The primary endpoint was the time to viral clearance. SARS‐CoV‐2 was detected by RT‐PCR using nasopharyngeal swabs on alternate days after hospital admission. If a test was positive for COVID‐19, the viral load was further quantified. A Ct value of >35 for both ORF1ab and N genes was considered negative. Negative nucleic acid conversion was defined as having two consecutive negative tests. The viral shedding time was defined as the interval between the first positive and first negative tests.

Following the Strengthening the Reporting of Observational Studies in Epidemiology (STROBE) guidelines, patient characteristics are expressed as median (interquartile range [IQR]) for continuous variables and n (%) for categorical variables. We performed a multivariable proportional odds model to explore the association between the COVID‐19 viral shedding time and age, while adjusting for sex, BMI, erythrocyte sedimentation rate (ESR), lymphocyte count, Ct values of the N gene, and CD8+ T lymphocyte count baseline confounders. We performed a redundancy analysis to ensure that no covariates completely explained any of the others (resulting in collinearity)^4^; no covariates suggested evidence of excessively high collinearity. A two‐tailed *p* < 0.05 was considered statistically significant. Statistical analysis was performed using the R software (version 4.2.1).

All patients provided written informed consent before enrollment in the study. The study protocol and informed consent forms were approved by the involved Ethics Committees, and the procedures accorded with the ethical standards of the responsible committee on human experimentation and the Helsinki Declaration of 1975, as revised in 1983.

## 3 RESULTS AND DISCUSSION

This study included 238 patients with mild COVID‐19, with an average age of 38 years (range: 23–52 years). Male patients accounted for 70.5% of the cases, and the median negative conversion time was 11 days (range: 8–15 days). Upon hospital admission, the average RT‐PCR Ct values for the ORF1ab and N genes, respectively, were 23 (range: 19–29.3) and 25 (range: 21–30.9). Comorbidities included hypertension (16%), diabetes (9%), chronic obstructive pulmonary disease (0%), and coronary atherosclerotic heart disease (6%). The mean BMI was 23.6 (range: 21.5–25.9) (Table 1).

**Table 1:** Demographic, clinical characteristics, and laboratory findings of Covid‐19 patients.

	Total *n* = 238	Male *n* = 168 (70.5%)	Female *n* = 70 (29.5%)
Duration, days	11 (8, 15)	12 (8, 16)	10 (7.2, 11)
Age, years	38 (23, 52)	40 (25, 53)	24 (21, 46)
BMI	23.6 (21.5, 25.9)	24.4 (22.3, 26.5)	22.5 (19.9, 24.2)
Hypertension (%)	38 (16%)	32 (19%)	6 (9%)
Diabetes (%)	22 (9%)	20 (12%)	2 (3%)
COPD (%)	0 (0)	0 (0)	0 (0)
CAD (%)	14 (6%)	14 (8%)	0 (0)
CT1	23 (19, 29.3)	22.8 (19.1, 29.4)	23.8 (18.8, 27.9)
CT2	25 (21, 30.9)	24.6 (21.1, 31)	26.3 (21, 30)
WBC (×10^9^/L)	5.4 (4.2, 7)	5.6 (4.3, 7)	4.6 (3.6, 6.5)
Plt (×10^9^/L)	208 (166, 260)	194 (159, 246)	233 (188, 268)
Plt/L	130 (101, 169)	127 (98, 167)	136 (105, 175)
Hb (g/L)	145 (134, 154)	150 (143, 155)	129 (122, 136)
N (×10^9^/L)	3.1 (2.2, 3.6)	3.3 (2.4, 4.7)	2.9 (2.1, 3.6)
L (×10^9^/L)	1.59 (1.18, 2.05)	1.58 (1.17, 2.04)	1.74 (1.23, 2.04)
hs‐CRP (mg/L)	2.35 (0.56, 4.59)	2.38 (0.55, 4.46)	1.94 (0.57, 4.59)
SSA (ng/mL)	10.6 (6.7, 30.8)	9.5 (6.3, 29.1)	17.6 (8.7, 33.8)
ESR (mm/h)	23 (8, 40.2)	20 (8, 33.2)	31 (9.2, 52.2)
ALT (U/L)	19 (14, 34)	23 (17, 37)	13 (11, 19)
AST (U/L)	20 (16, 26)	20 (18, 28)	18 (15, 21)
Scr (g/L)	72.7 (61.1, 85.1)	80.2 (71.5, 88.8)	57.1 (51.5, 62.7)
Alb (g/L)	43 (41, 45)	43 (40.9, 45)	43 (41, 45)
PA (g/L)	237 (202, 268)	243 (204, 267)	221 (198, 268)
Uacid (g/L)	334 (266, 390)	363 (308, 410)	260 (238, 305)
d‐dim (mg/L)	0.21 (0.17, 0.32)	0.2 (0.17, 0.29)	0.27 (0.17, 0.41)
IL‐6 (ng/mL)	1.4 (0.77, 2.66)	1.4 (0.77, 2.79)	1.13 (0.65, 2.59)
IL‐8 (ng/mL)	0.34 (0, 8.3)	0.48 (0, 9.26)	0.13 (0, 3.66)
CD3 (numbers)	1051 (747, 1428)	1036 (782, 1414)	1140 (715, 1491)
CD8 (numbers)	426 (269, 598)	411 (267, 574)	465 (290, 632)
CD4 (numbers)	590 (388, 817)	590 (390, 795)	590 (392, 844)

*Note*: Data are median (IQR) or *n* (%). CD3/CD4/CD8 = CD3+ T cell, CD4+ T cell, CD8+ T cell.

Abbreviations: Alb, albumin; ALC, absolute lymphocyte count; ALT, alanine transaminase; AST, aspartate aminotransferase; BMI, body mass index; CAD, cardiovascular disease; COPD, chronic obstructive pulmonary disease; CT1, cycle threshold values of ORF1ab; CT2, cycle threshold values of N gene; d‐dim, d‐Dimer; ESR, erythrocyte sedimentation rate; Hb, hemoglobin; hs CRP, high sensitivity C‐reactive protein; IL‐6, interleukin‐6, IL‐8, interleukin‐8; IQR, interquartile range; L, lymphocyte; N, neutrophil; N/L, neutrophil 1ymphocyte ratio; PA, prealbumin; Plt, platelet, Plt/l, platelet lymphocyte ratio; SAA, serum amyloid A; Scr, serum creatinine; uacid, serum uric acid; WBC, white blood cell.

**Figure 1:**
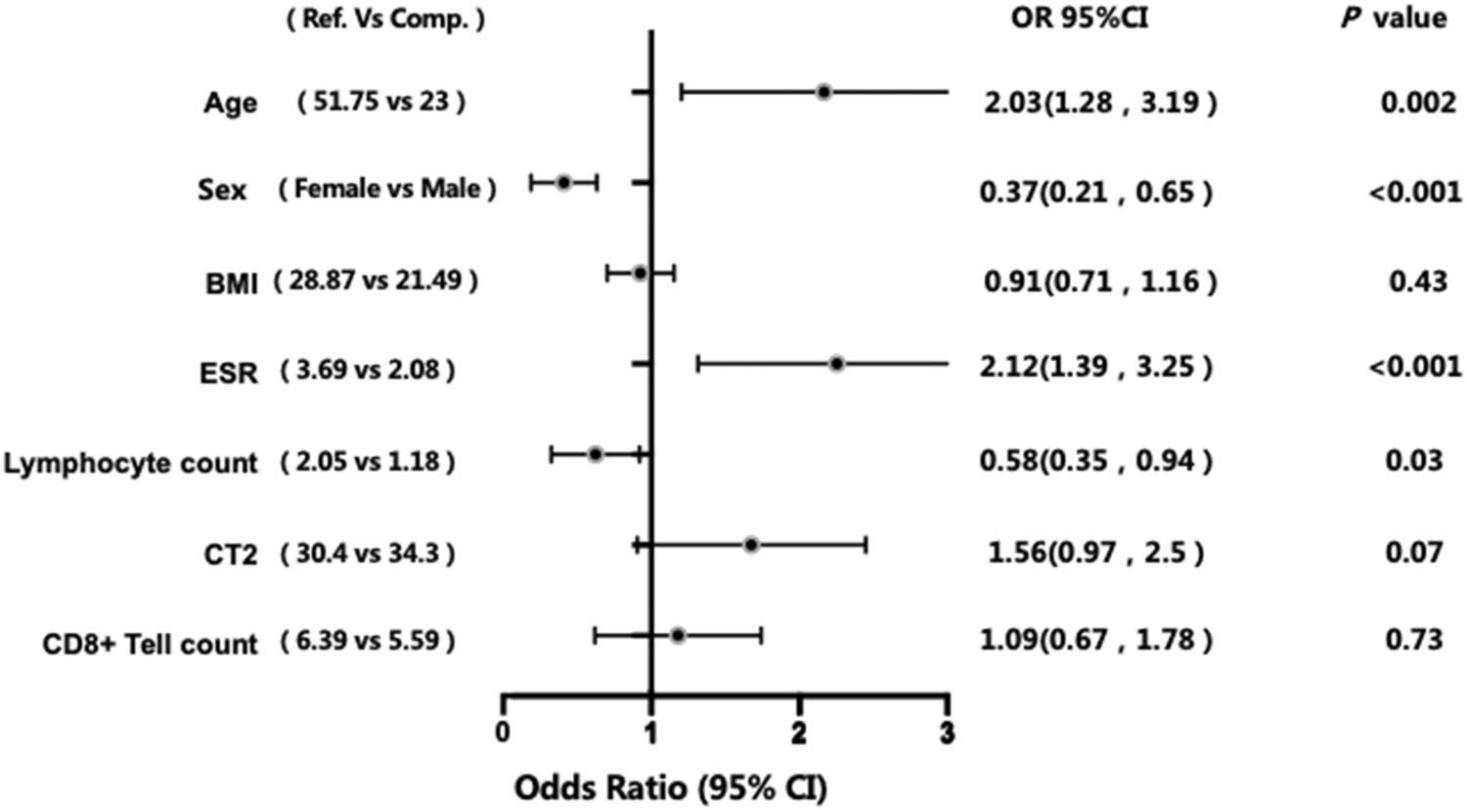
Forest plot of probability of duration of virus shedding. BMI, body mass index; CI, confidence interval; ESR, erythrocyte sedimentation rate.

Risk association analysis between median follow‐up period as a categorical variable and negative conversion time in subgroups of age, sex, BMI, ESR, ALC, CT2, and CD8+ T cell count was performed. The median follow‐up period was 11 days (IQR: 8–15 days). Age (51.75 vs. 23; odds ratio [OR] = 2.03; 95% confidence interval [CI]: 1.28–3.19, *p* = 0.002), sex (women vs. men; OR = 0.37; 95% CI: 0.21–0.65; *p* < 0.001), peripheral absolute lymphocyte count (ALC; 2.05 vs. 1.18; OR = 0.58; 95% CI: 0.35–0.94; *p* = 0.03), and ESR (OR = 2.12; 95% CI: 1.39–3.25; *p* < 0.001) were independent predictors of negative conversion time. Differences were considered statistically significant when the *p*‐value was <0.5 (Figure 1).

Age, sex, ALC, and ESR were identified as independent risk factors affecting the negative conversion time. For example, the negative conversion time increased with age. This may result from age‐related immune deterioration. Meanwhile, the incidence of comorbidities—including hypertension, coronary heart disease, chronic obstructive pulmonary disease, and diabetes—increases with age, potentially increasing susceptibility to viral infection and prolonged negative conversion times after infection.^5^ Notably, age predicts the conversion rate and mortality of critically ill COVID‐19 patients.^6^, ^7^

The results also indicated that male patients had longer nucleic acid conversion times. Studies have shown that male COVID‐19 patients, especially older male patients, have higher rates of hospitalization, severe illness, and mortality than female patients, suggesting a possible influence of sex hormones on the function of immune cells. Indeed, in terms of adaptive immune response, women generally exhibit stronger humoral and cell‐mediated immune responses against antigenic stimulation, vaccination, and infection.^8^, ^9^ Most genes with sex‐differential expression were significantly upregulated in the B cells of adult women compared to men.^10^ Clinical studies have also revealed that men have lower CD3+ and CD4+ cell counts, CD4+:CD8+ cell ratios, and helper T cell type 1 responses as compared with women.^11^ Cytotoxic T cell activity, as well as the expression of antiviral and proinflammatory genes, is higher in women, and many of the relevant genes contain estrogen‐responsive elements in their promoters.^12^

Similarly, our results suggest that a lower ALC prolongs patients' nucleic acid conversion time. Lymphocytes reflect immune status, and in patients with severe COVID‐19, a high lymphocyte count indicates a better clinical prognosis.^13^ This study on patients with mild COVID‐19 found that the basic CD3+ , CD4+ , and CD8+ levels were within normal range, indicating that the patient's immune system was in a normal state. Meanwhile, a highly deregulated immune response may result in excessive inflammation,^14^ increased levels of IL‐6 and IL‐8, and even increased risk of death, that is, progression from mild to critical illness.^15^ However, herein, no patients experienced critical inflammation initially or during follow‐up, and the cytokines (IL‐6 and IL‐8) were within the normal range.

Patients' ESR was also positively correlated with the negative conversion time. Indirectly, ESR indicates both acute phase response and acute inflammatory response markers (together with fibrinogen). Herein, a relatively high ESR was associated with mortality from COVID‐19. Moreover, several studies included ESR as a prognostic indicator, with its clinical significance directed toward the inflammatory response.^16^, ^17^

The median Ct values for the N and ORF1ab genes in the RT‐PCR assay were 23 and 25, respectively, on the admission day, and showed no significant correlation with the negative conversion time. Conversely, previous studies have suggested that these values are indirectly related to viral load, with higher values possibly predicting shorter negative conversion time.^18^ The discrepancy observed here suggests that Ct values may variably predict the negative conversion time in different studies, depending on the included variables.

This study has several limitations. First, the sample size was small. Second, stratified analyses should be conducted based on RT‐PCR‐derived Ct values and underlying disease conditions. Furthermore, the relatively small number of events may have affected the number of variables included in the logistic regression analysis.

## AUTHOR CONTRIBUTIONS

**Hai Zou**: conceptualization; data curation; formal analysis; funding acquisition; supervision; writing – original draft; writing – review & editing. **Jun Zhang**: conceptualization; supervision. **Wencong Chen**: data curation; supervision; validation. **Xinyan Li**: conceptualization; supervision. **Biao Zhu**: formal analysis; software; supervision.

## CONFLICTS OF INTEREST STATEMENT

The authors declare no conflicts of interest.

## ETHICS STATEMENT

Written informed consents were obtained from all patients before enrollment in the study. The study protocol and informed consent forms were approved by the involved Ethics Committees, and the procedures followed were in accordance with the ethical standards of the responsible committee on human experimentation and with the Helsinki declaration of 1975, as revised in 1983.

## TRANSPARENCY STATEMENT

The lead author affirms that this manuscript is an honest, accurate, and transparent account of the study being reported; that no important aspects of the study have been omitted; and that any discrepancies from the study as planned (and, if relevant, registered) have been explained.

## Data Availability

All data relevant to the study are included in the article.
